# Copper pillar and memory characteristics using Al_2_O_3_ switching material for 3D architecture

**DOI:** 10.1186/1556-276X-9-366

**Published:** 2014-07-26

**Authors:** Siddheswar Maikap, Rajeswar Panja, Debanjan Jana

**Affiliations:** 1 Thin Film Nano Tech. Lab., Department of Electronic Engineering, Chang Gung University, 259 Wen-Hwa 1st Rd, Kwei-Shan, Tao-Yuan 333, Taiwan

**Keywords:** Resistive memory, Cu pillar, 3D cross-point architecture, High density

## Abstract

A novel idea by using copper (Cu) pillar is proposed in this study, which can replace the through-silicon-vias (TSV) technique in future three-dimensional (3D) architecture. The Cu pillar formation under external bias in an Al/Cu/Al_2_O_3_/TiN structure is simple and low cost. The Cu pillar is formed in the Al_2_O_3_ film under a small operation voltage of <5 V and a high-current-carrying conductor of >70 mA is obtained. More than 100 devices have shown tight distribution of the Cu pillars in Al_2_O_3_ film for high current compliance (CC) of 70 mA. Robust read pulse endurances of >10^6^ cycles are observed with read voltages of −1, 1, and 4 V. However, read endurance is failed with read voltages of −1.5, −2, and −4 V. By decreasing negative read voltage, the read endurance is getting worst, which is owing to ruptured Cu pillar. Surface roughness and TiO_*x*_N_*y*_ on TiN bottom electrode are observed by atomic force microscope and transmission electron microscope, respectively. The Al/Cu/Al_2_O_3_/TiN memory device shows good bipolar resistive switching behavior at a CC of 500 μA under small operating voltage of ±1 V and good data retention characteristics of >10^3^ s with acceptable resistance ratio of >10 is also obtained. This suggests that high-current operation will help to form Cu pillar and lower-current operation will have bipolar resistive switching memory. Therefore, this new Cu/Al_2_O_3_/TiN structure will be benefited for 3D architecture in the future.

## Background

**Figure 1:**
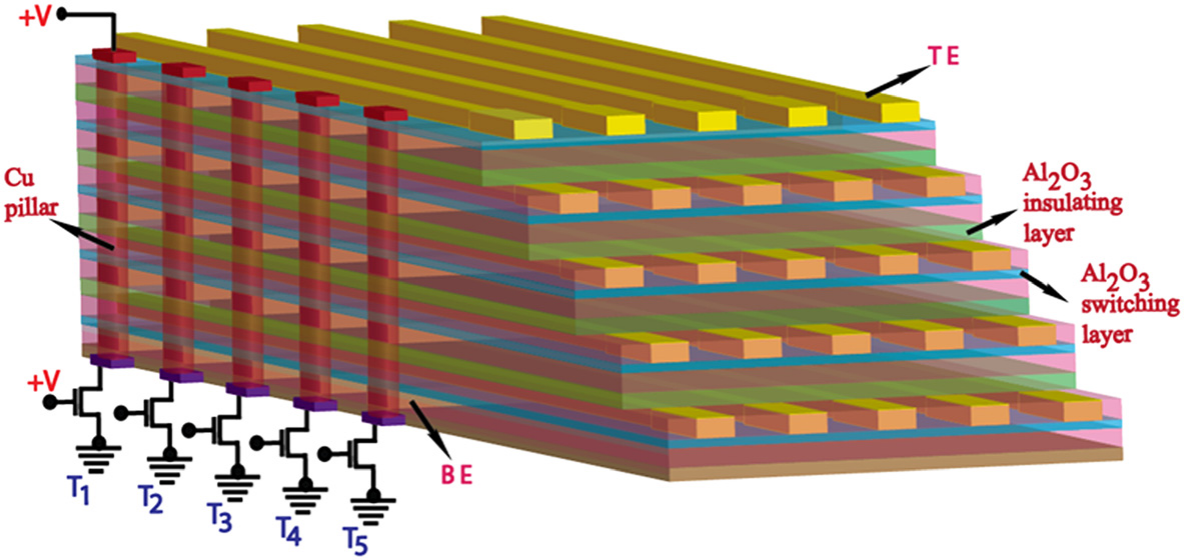
**Proposed 3D cross-point architecture by using Cu pillar.** Schematic view of proposed three-dimensional cross-point architecture with copper (Cu) pillar for high-density memory application. It is expected that five layers of cross-point RRAM devices will be connected by using Cu pillar through Al_2_O_3_ isolation layer because Cu could be migrated through Al_2_O_3_ film under external positive bias on the TE. This is the general theory from conductive bridging resistive random access memory (CBRAM) devices.

Recently, resistive random access memory so-called RRAM has attracted great attention to the researchers owing to its simple metal-insulator-metal (M-I-M) structure, long endurance, low-power consumption, good data retention, and excellent scalability [1-5]. To observe the acceptable resistive switching behavior, some switching materials such as TaO_*x*_[6-8], HfO_*x*_[9,10], and AlO_*x*_[11-13] show promise for future applications. Further, to obtain high-density and device scaling, different kinds of device structures have been reported [14-16]. Ho et al. [14] have fabricated a 9-nm half-pitch RRAM device using WO_*x*_ material. Chen et al. [15] has fabricated a 10 × 10 nm^2^ cross-point device using HfO_*x*_ material. Kim et al. [16] has demonstrated a RRAM device with ‘dash BE’ having an effective device area of 5 nm × 65 nm using Pt electrodes for TaO_*x*_-based material. However, RRAM suffers to replace mainstream conventional FLASH memory even though it exhibits good scalability and high speed operation (few ns). Many challenges need to be overcome. One of the challenges of RRAM is to improve the integration density which can also compete with conventional FLASH in market. In recent days, the flash technology approaches its scaling limit in sub-20-nm regime and as an alternative, three-dimensional (3D) stackable NAND flash is feasible by using through-silicon-vias (TSV) method [17,18]. To obtain the similar device density as the product 3D flash, the 3D scalable (<20 nm) RRAM is necessary in the future which is demonstrated in literature rarely [19-21]. Yu et al. [19] and Chien et al. [20] have reported sidewall RRAM memories using HfO_*x*_ and WO_*x*_ materials, respectively. Kügeler et al. [21] have reported resistive switching effect in high-density 3D cross-point architecture using AlO_*x*_ material. Basically, the cross-point memory devices have been reported by several groups. However, there is no report on interconnection of 3D architecture of RRAM, which is one of the bottlenecks to reach high-density memory application. Therefore, a novel approach to form Cu pillar in the Al_2_O_3_ material has been investigated for the first time. A simple M-I-M structure can be transferred in the 3D cross-point architecture with Cu pillar for high-density, low-energy, and low-cost applications. By applying a positive voltage which is larger than the set voltage, the Cu pillar in an Al/Cu/Al_2_O_3_/TiN structure could be formed due to the migration of Cu ions and make contact from one stack to another stack as shown in Figure 1. The Cu migration has a similar function with conductive bridging resistive random access memory (CBRAM). The Cu pillar diameter will be controlled through current limit of series transistor (T1-5), and this transistor will be used to control also the current compliance of RRAM or CBRAM devices. To obtain 3D stack, the chemical–mechanical-polishing (CMP) will be used after Al_2_O_3_/BE (and/or Al_2_O_3_/TE) step. Due to this Cu pillar formation, the area consumed by cross-points will be lesser than that of the conventional cost-effective TSV method. It is well known that the TSV is used for 3D architecture. However, it has a high cost and still needs a larger area. To get a low-cost and high-density Cu interconnection for 3D stacks, 3D architecture with Cu pillar would be a good alternative to overcome the aforementioned TSV issue [22]. In this cross-point architecture (Figure 1), the Cu as an oxidize electrode or top electrode (TE) could be used; other inert electrodes such as tungsten (W) and titanium-nitride (TiN) or bottom electrode (BE) could be used; and Al_2_O_3_ film could be used as switching layer. The Al_2_O_3_ film as a resistive switching material is very promising for future applications [10-13]. This new idea of 3D RRAM using Cu pillar with Al_2_O_3_-based resistive switching memory is reported for the first time.

To succeed the 3D memory architecture with Cu pillar in the future, the via-hole with a size of 4 × 4 μm^2^ was fabricated in an Al/Cu/Al_2_O_3_/TiN M-I-M structure in this study. Tight distribution of the Cu pillars for 100 devices is observed with a low formation voltage of <5 V and high current compliance (CC) of 70 mA. The formation of strong metallic path in Al_2_O_3_ layer suggests that Cu pillar could be formed. The Cu pillars have long read pulse endurance of >10^6^ cycles under positive read voltage; however, it has short read endurance under negative read voltages of less than −1.5 V, owing to random read stress-dependent ruptured Cu pillar. On the other hand, bipolar resistive switching memory characteristics are observed by reducing the CC of 500 μA under a small operating voltage of ±1 V. The resistive switching mechanism is formation/dissolution of Cu filament in the Al_2_O_3_ film under external bias. The memory device has good data retention of >10^3^ s with acceptable resistance ratio of >10.

## Methods

**Figure 2:**
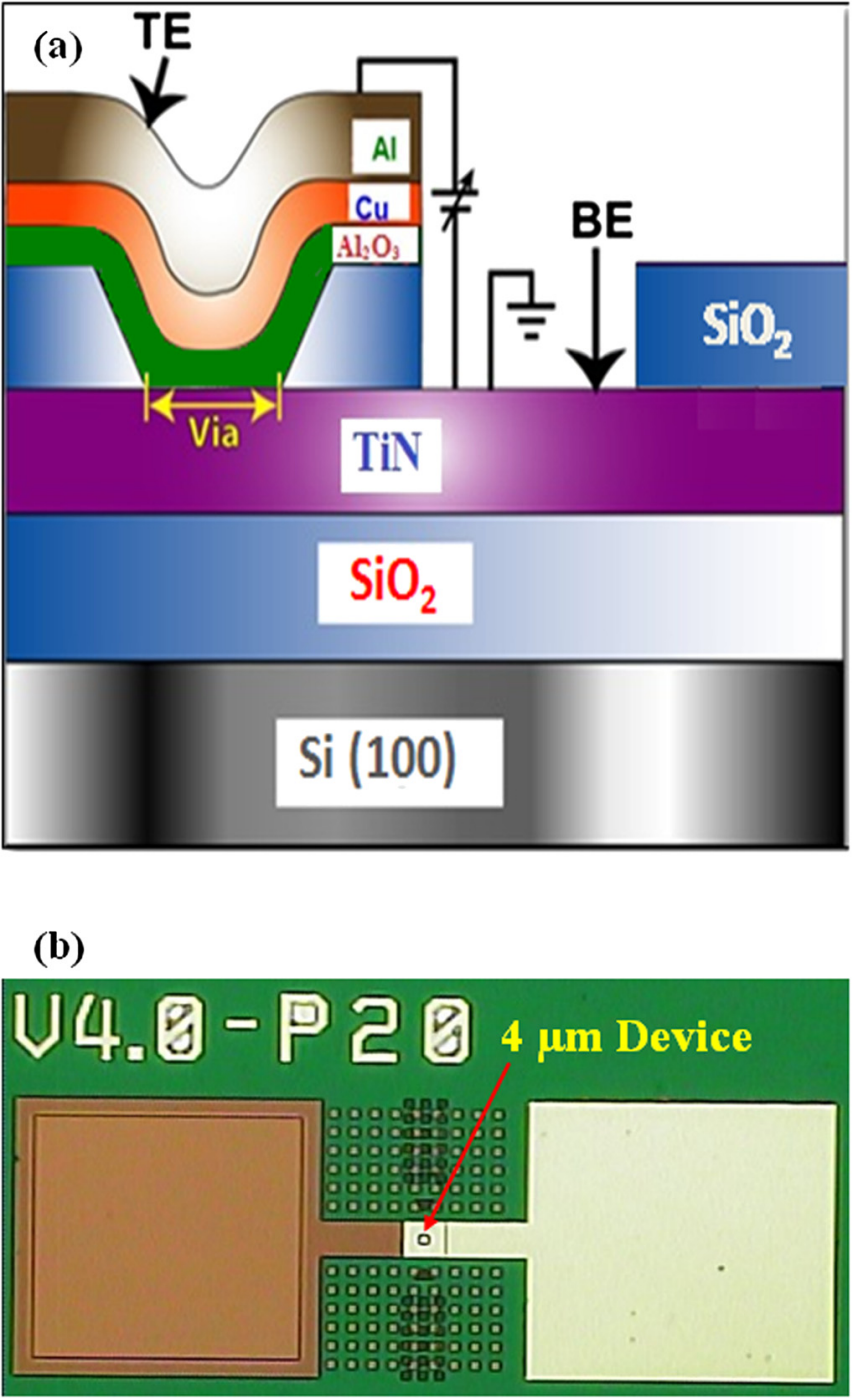
**Schematic view of via-hole device and OM image. (a)** Schematic view of the Cu pillar formation and memory characteristics of an Al/Cu/Al_2_O_3_/TiN structure. **(b)** Optical image (OM) of a typical 4 × 4 μm^2^ device. The ‘V4.0’ as indicated on OM image is via size of 4 × 4 μm^2^.

**Figure 3:**
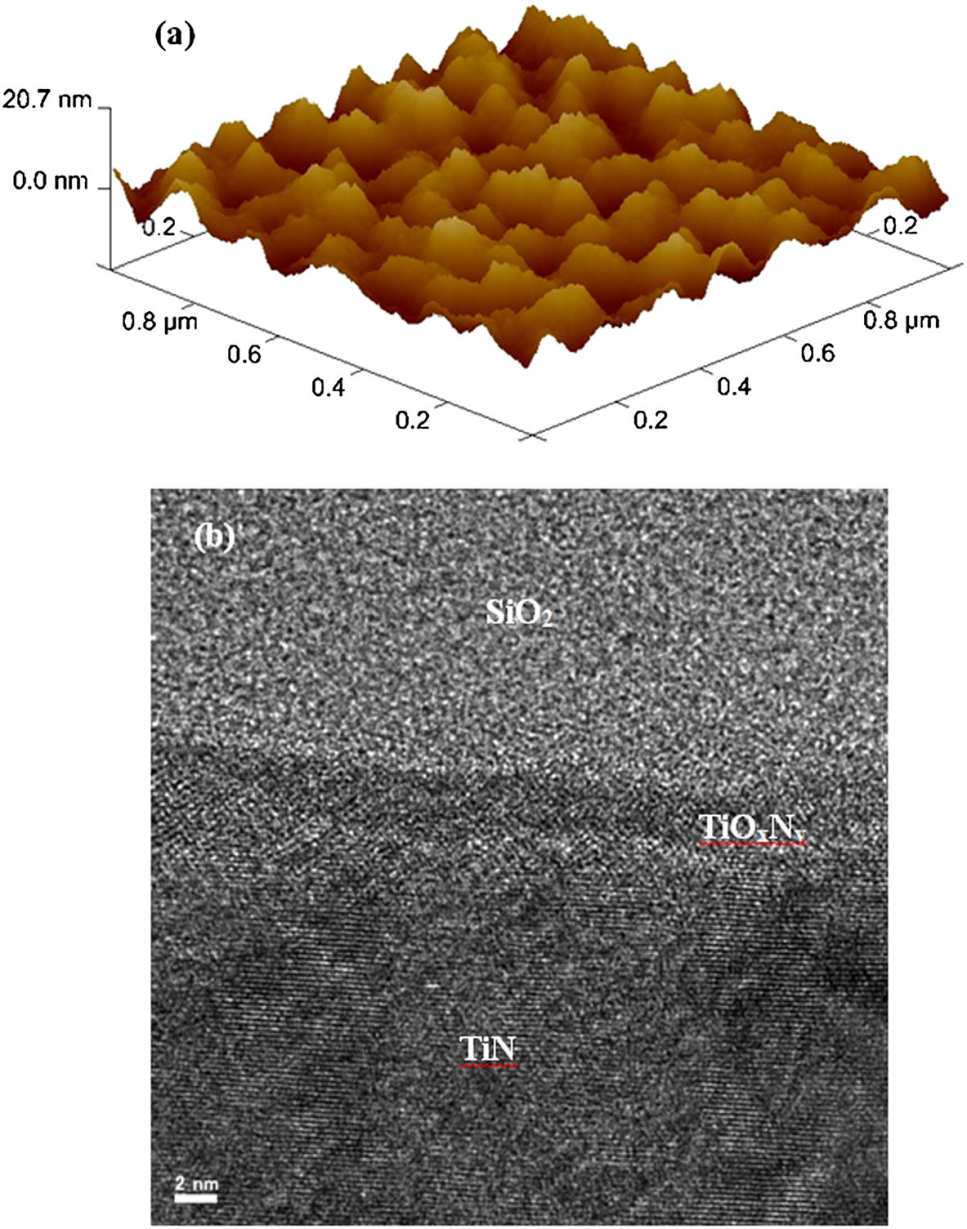
**AFM and HRTEM images for TiN layer. (a)** Atomic force microscope (AFM) image shows surface roughness of TiN layer with a scan area of 1× 1 μm^2^. **(b)**The TiN surface is oxidized and is observed by high-resolution transmission electron microscope (HRTEM) image.

Titanium-nitride (TiN) as a bottom electrode (BE) was deposited on 8-in. SiO_2_ (200 nm)/Si substrates. The thickness of TiN BE was approximately 200 nm. Then, the SiO_2_ film with a thickness of 150 nm was deposited. The via-holes with a size of 4 × 4 μm^2^ were patterned by lithography and opened by dry etching. To follow the lift-off process, photo-resist (PR) was coated and opened on the via-hole and top electrode (TE) regions. Then, the Al_2_O_3_ switching layer with a thickness of approximately 20 nm was deposited by rf sputtering. The Al_2_O_3_ target with a purity of 99.9% was used for deposition. During deposition, the argon (Ar) flow rate was 25 sccm. The deposition power and pressure was 80 W and 30 mTorr, respectively. In next step, Cu as a TE was deposited by thermal evaporator. The deposition rate was 0.5 Å/s. The thickness of Cu was approximately 40 nm. After that aluminum (Al) as a capping layer was deposited by using the same thermal evaporator. The Al deposition rate was 1 Å/s. The thickness of Al was approximately 160 nm. Finally, lift-off was performed to get the final resistive switching memory device. The schematic view of our Al/Cu/Al_2_O_3_/TiN via-hole device is shown in Figure 2a. Optical microscope image of the via-hole with a size of 4 × 4 μm^2^ is shown in Figure 2b. Both the TE and BE were also isolated from other devices. The surface roughness of the BE was observed by the atomic force microscopy (AFM) image in 3D (dimension), as shown in Figure 3a. The images were taken in tapping mode from Innova Scanning Probe Microscope (SPM) system. The average and root mean square (RMS) roughness values were found to be 2.66 and 3.28 nm, respectively. However, the TiN surface was oxidized and it became TiO_*x*_N_*y*_. The surface of TiN Be was also observed by transmission electron microscope (TEM, JEOL 2100 F, JEOL Ltd., Akishima-shi, Japan) with energy of 200 keV, as shown in Figure 3b. The thickness of TiO_*x*_N_*y*_ layer was approximately 3.5 nm. During electrical measurement, the bias was applied on the Cu TE while the BE was grounded. All the electrical measurements were carried out by Agilent 4156C semiconductor parameter analyzer (Agilent Technologies, Inc., Santa Clara, CA, USA).

## Results and discussion

**Figure 4:**
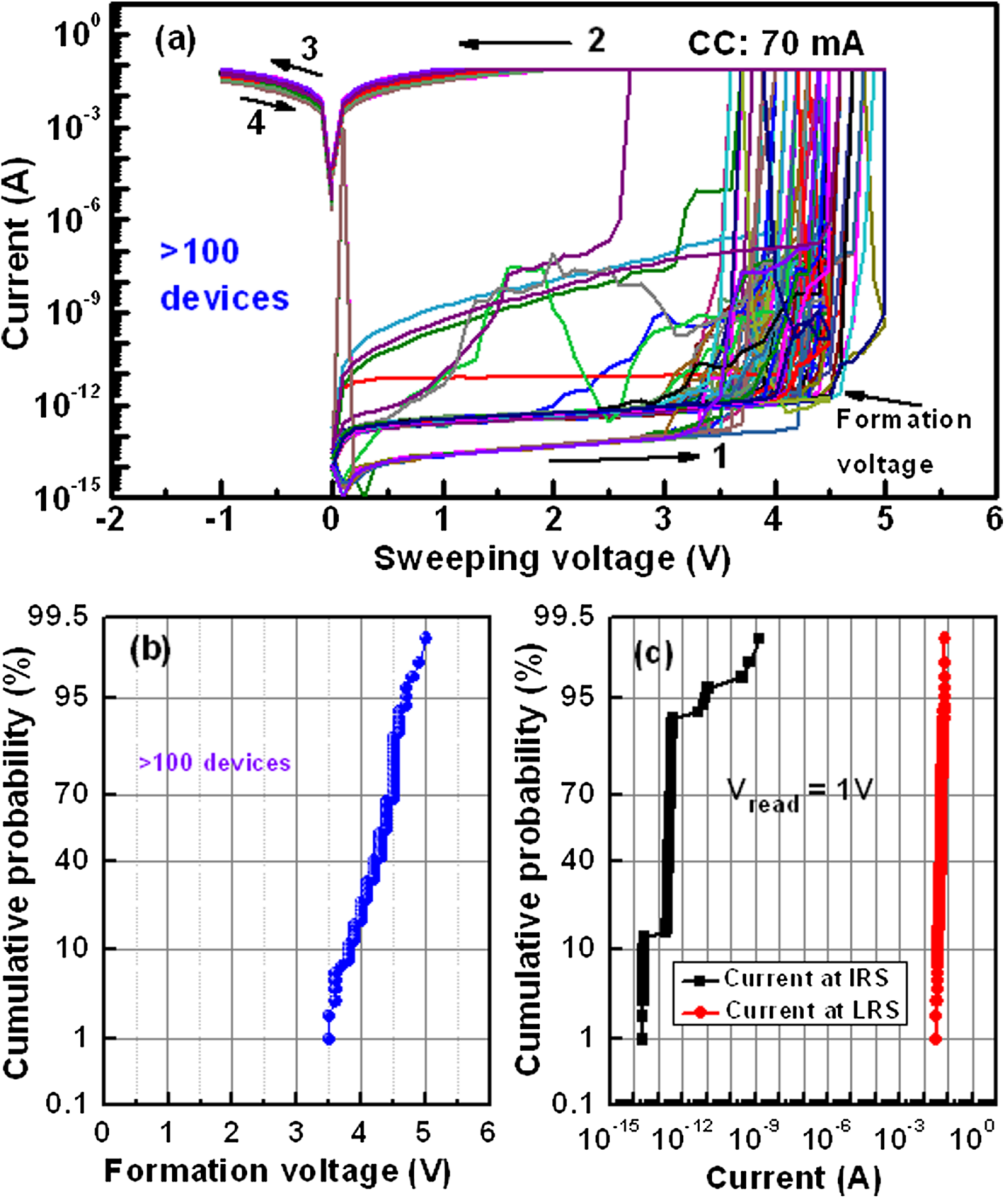
**Current–voltage (I-V) characteristics and statistical distribution. (a)** Current–voltage (I-V) characteristics of randomly measured 100 devices at a high CC of 70 mA. Statistical distribution of **(b)** forming voltage, **(c)** current levels at IRL and LRS for the Al/Cu/Al_2_O_3_/TiN CBRAM devices.

Figure 4a shows current–voltage (I-V) characteristics of randomly measured 100 pristine devices in an Al/Cu/Al_2_O_3_/TiN structure. The sweeping voltages (0 → +5 → 0 → −1 → 0 V) applied on the TE is shown by arrows 1 to 4. A high current compliance of 70 mA is reached. Initial resistance state (IRS) shows high because of insulating properties of the Al_2_O_3_ film. After applying positive formation voltage (*V*_form_) on the TE, the device switches from IRS to low-resistance state (LRS). If current compliance is higher than 75 mA, then some devices are burned out because of joule heating. That is why the current compliance of 70 mA was used to protect the device. These devices do not show reset operation even a reset voltage of −1 V. This suggests that the strong Cu filament or pillar forms in the Al_2_O_3_ film, which we are looking at the metal interconnection for 3D memory stack. Figure 4b represents the narrow distribution of V_form_ for the 100 device-to-devices. The read voltage was 1 V. The mean value (*σ*_m_) and standard deviation (*σ*_s_) of forming voltages are +4.25 V and 0.3491. This implies that small external voltage (<5 V) is needed to form Cu pillar. Almost all devices have the formation of Cu pillar, which suggests the 100% yield. To analyze the device-to-device uniformity, both currents of IRS and LRS were read (*V*_read_) at a voltage of +1 V (Figure 4c). The *σ*_m_ values of currents at IRS and LRS are found to be 25.9 pA and 49.96 mA, whereas the standard deviation (*σ*_s_) are 172.19 and 9.33, respectively. At *V*_read_ of +2 V, the current through Cu pillar is 70 mA. This indicates that the current at LRS for the 100 devices follows the same current path. Tight distribution of high current at LRS indicates that strong Cu pillars are formed to connect each stack in 3D cross-point architecture for high-density memory application. This Cu pillar should be a good alternative of conventional TSV for 3D integrated circuit (IC) interconnection because of a simple process and cost-effectiveness.

**Figure 5:**
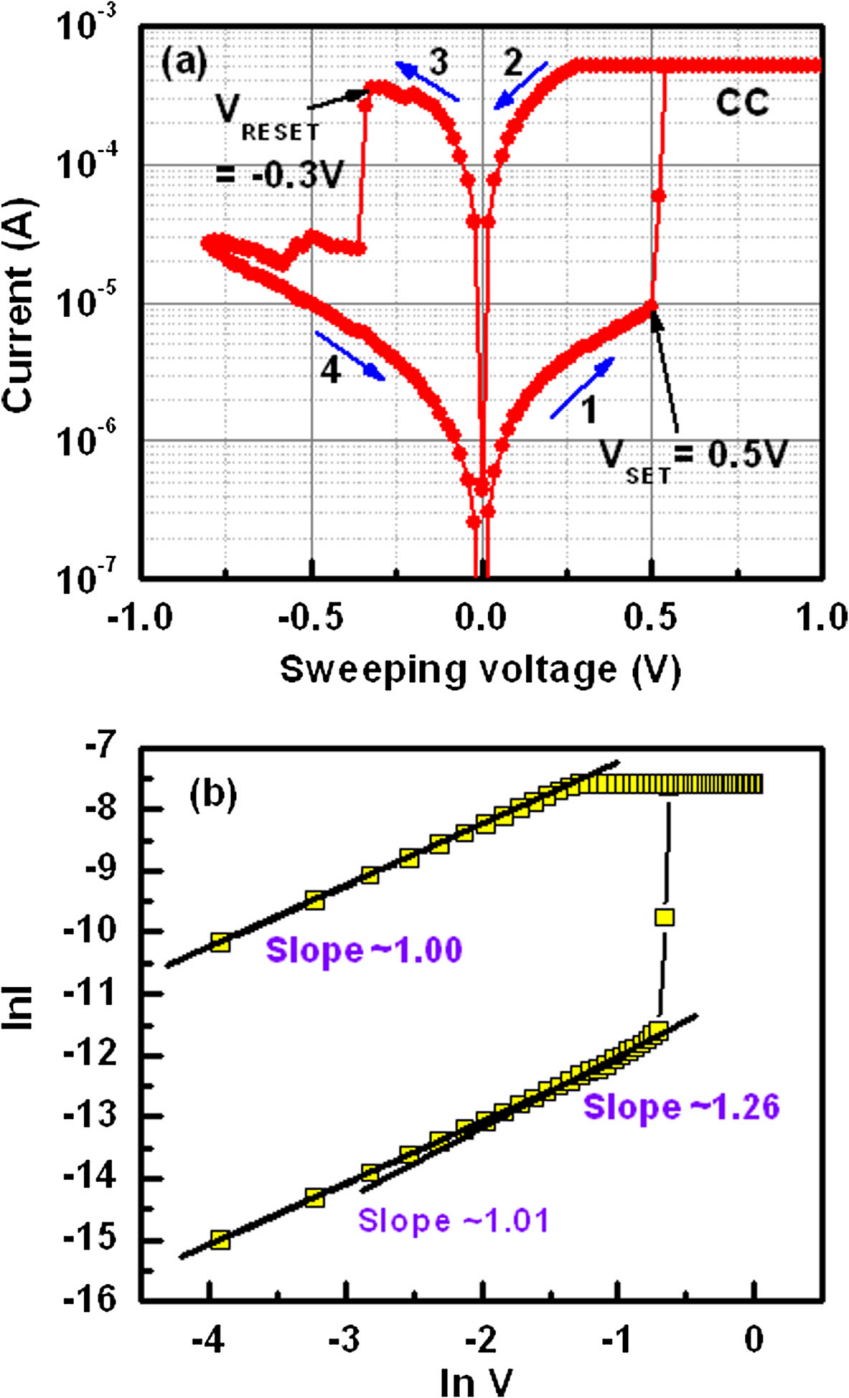
**I-V characteristics and conduction mechanism. (a)** Bipolar resistive switching characteristics of the Al/Cu/Al_2_O_3_/TiN memory device at a CC of 500 μA under small operating voltage of ±1 V is observed. **(b)** To identify the current conduction mechanism, I-V curves are fitted in log-log scale. Both HRS and LRS show ohmic current conduction behavior.

**Figure 6:**
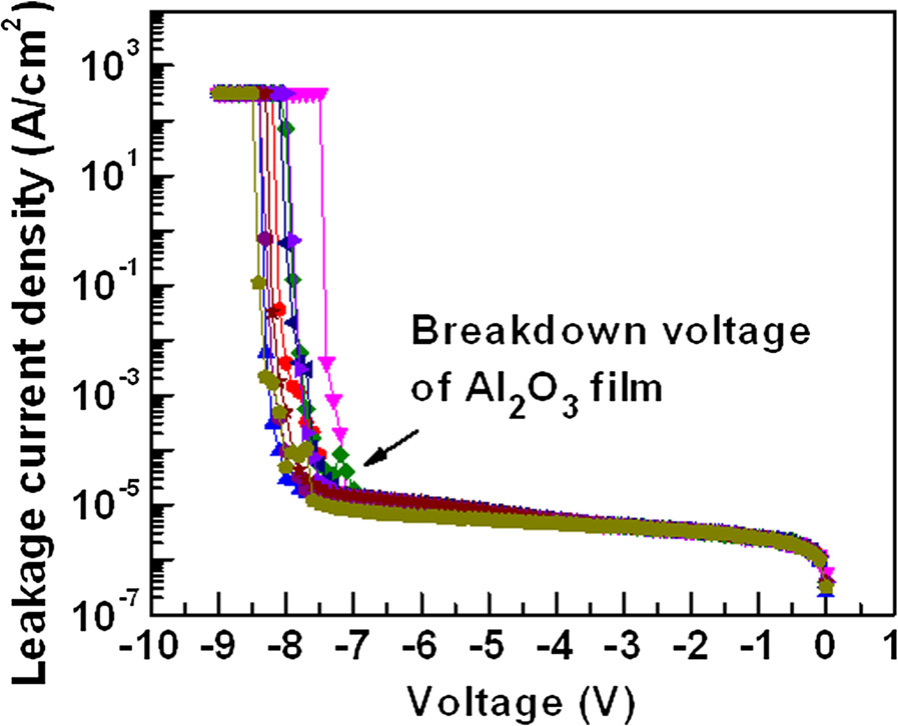
**Breakdown voltage characteristics of Al**_**2**_**O**_**3**_**layer.** The magnitude of negative breakdown voltage is higher than that of the positive-formation voltage. This suggests that Cu migration through the Al_2_O_3_ layer is observed under positive bias on the TE.

Figure 5a shows bipolar resistive switching characteristics at a low CC of 500 μA for the Al/Cu/Al_2_O_3_/TiN CBRAM devices. After formation and first reset operation, the arrows (1 → 4) indicate the direction of I-V sweep (0 → +1 → 0 → −0.8 → 0 V). Therefore, low operation voltage of +1 to −0.8 V is needed. The set voltage (*V*_SET_) is about 0.5 V and reset voltage (*V*_RESET_) is −0.3 V. The reset current of ~400 μA is lower than the compliance current. The currents at HRS and LRS are 1.5 and 190 μA at *V*_read_ of 0.1 V. A good resistance ratio of approximately 130 is obtained. The switching mechanism is based on the formation and dissolution of Cu metallic filament depending on electrical stimulus of our Al/Cu/Al_2_O_3_/TiN memory devices. When the positive bias is applied on the TE, the Cu ions will be migrated through the Al_2_O_3_ film and form Cu metallic path in between TE and BE by reduction process (Cu^*z*+^ + *z*e^−^ → Cu^o^, where *z* is 1 to 2). When the negative bias (−Ve) is applied on the TE, the Cu metallic filament will be dissolved into the Al_2_O_3_ film by oxidation process (Cu^o^ → Cu^*z*+^ + *z*e^−^). The Cu filament reduction/oxidation was also observed in our previous work by using different materials such as TaO_*x*_[7] and GeO_*x*_[23]. Two step *V*_RESET_s are observed in this study. First, the filament is dissolved at −0.3 V. Second, the remaining filament is dissolved at −0.5 V. However, by applying further negative voltage on the TE, the Al_2_O_3_ film will be breakdown or re-growth of Cu filament [23] could be observed because of the remaining Cu material on the BE. Therefore, the magnitude of negative bias is sensitive to control the resistive switching properly. The Cu ion migration is also confirmed by measuring the breakdown voltage of the Al_2_O_3_ film in the Al/Cu/Al_2_O_3_/TiN pristine devices. Figure 6 shows the breakdown characteristics of the Al/Cu/Al_2_O_3_/TiN devices. Randomly, 10 devices were measured. The value of breakdown voltage is higher as compared to positive-forming voltage (−7 to −8 V vs. 3.5 to 5 V). By applying negative voltage, the Cu ions are not migrated through the Al_2_O_3_ films; however, higher negative voltage is required to break the Al-O bonds to form the oxygen vacancy conducting path. On the other hand, electrochemically active Cu ions migrate through Al_2_O_3_ layer under smaller positive voltage (<5 V), which will form the metallic conducting paths. To identify the current conduction mechanism of the CBRAM devices, I-V curve fitted in log-log scale, as shown in Figure 5b. Slope value of LRS is 1 (IαV) whereas slope values of HRS are 1.01 (IαV^1.01^) at low voltage region and 1.26 (IαV^1.26^) at high-voltage regions. This suggests that conduction mechanism of both LRS and HRS exhibits ohmic current conduction behavior. LRS is ohmic owing to Cu metallic path formed in the Al_2_O_3_ film. On the other hand, when we apply negative bias on the TE, the Cu metallic path in the Al_2_O_3_ film is partially dissolved; the rest of the part is metallic path; and Cu metals remain in the Al_2_O_3_ film. This causes also the ohmic conduction behavior at HRS.

**Figure 7:**
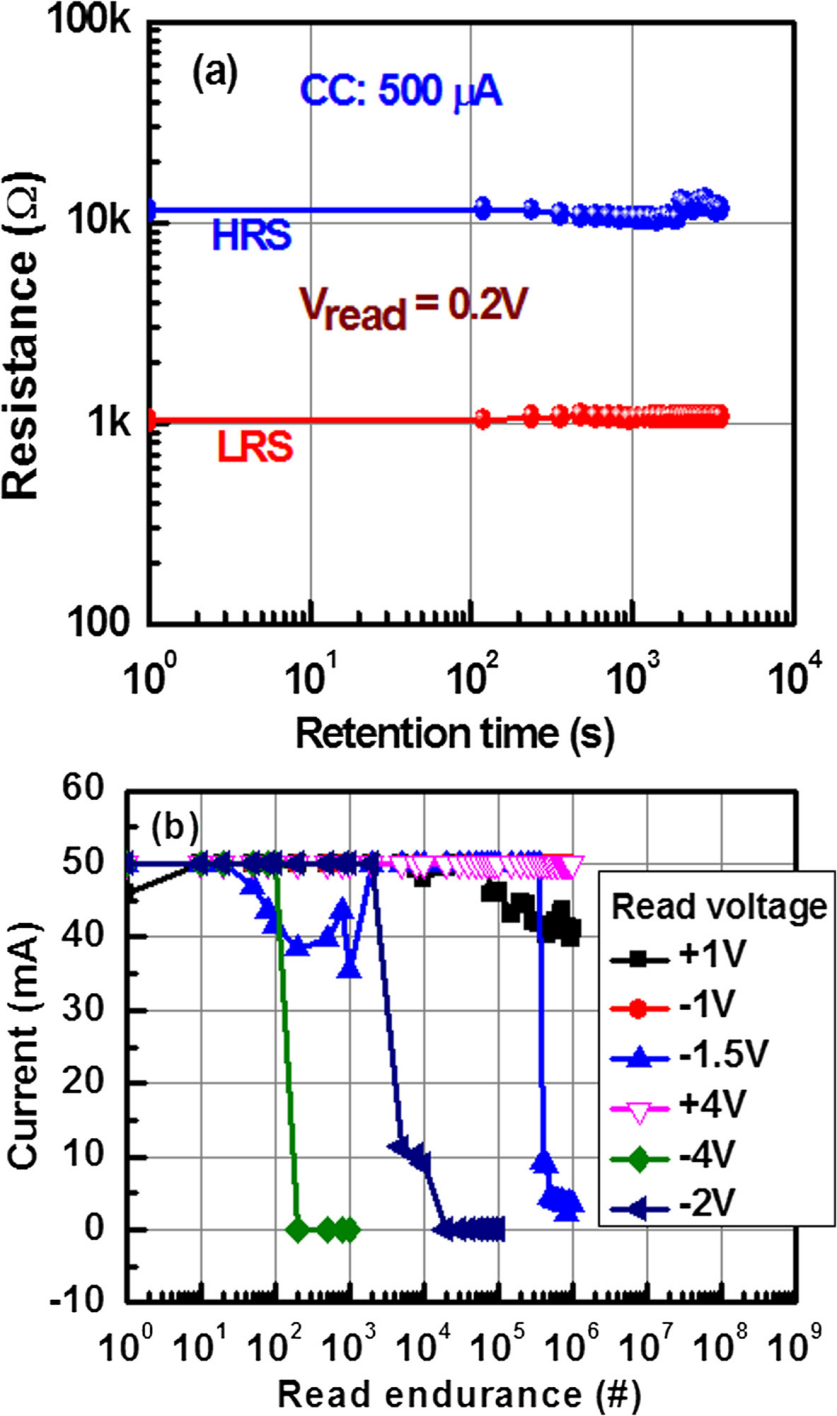
**Data retention and read endurance characteristics. (a)** Typical data retention characteristics of our Al/Cu/Al_2_O_3_/TiN CBRAM device. The thickness of Al_2_O_3_ layer is 10 nm. **(b)** Read endurance characteristics of the Cu pillars in a Al/Cu/Al_2_O_3_/TiN structure at high CC of 70 mA. The stronger Cu pillars are obtained when the bias is positive.

Figure 7a shows good data retention characteristics of >10^3^ s at CC of 500 μA. After 10^3^ s, memory device maintains >10 resistance ratio, which is acceptable for future non-volatile memory application. Figure 7b represents the read endurance characteristics of the Cu pillars in the Al/Cu/Al_2_O_3_/TiN M-I-M structures. After applying high CC of 50 mA on the pristine devices, we check the read endurance characteristics of LRS at different positive and negative read voltages of +1, +4, −1, −1.5, −2, and −4 V accordingly. The Cu pillars have robust read endurances of >10^6^ cycles with no degradation under *V*_read_ of +1, +4, and −1 V accordingly. The stress pulse width is 500 μs and read pulse width is 10 ms. At *V*_read_ of +1 V, initial read current is 50 mA. The current decreases slightly to approximately 40 mA after 10^6^ cycles. This indicates that some weak Cu filaments are broken during read pulse endurance at a high value of negative voltage. At *V*_read_ of +4 V, the Cu pillars are stronger (>10^6^ cycles) because Cu could be diffused under high positive voltage on the TE. Even at *V*_read_ of −1 V, the longer and stable read endurance is observed. This suggests that the Cu pillar is not dissolved with a negative voltage of −1 V on the TE. However, failure of read cycles with increasing negative voltage is observed. The read cycles of approximately 350,000, 2,000, and 100 are observed with *V*_read_ of −1.5, −2, and −4 V, respectively. This suggests that the Cu pillar is ruptured under a lower voltage of less than −1.5 V, and it is owing to joule heating by random stress. This suggests that reliable Cu pillar is observed with operation voltage of larger than −1 V, and it is destroyed with operation voltage of lower than −1.5 V. It seems that the resistive switching memory device can be programmed under positive voltage through Cu pillar; however, it is not possible to erase through Cu pillar if it needs lower voltage than that of −1.5 V. Further study is needed to improve Cu pillar robustness under negative voltage on the Cu electrode.

## Conclusions

The Cu pillars are formed in Al/Cu/Al_2_O_3_/TiN structure under a small voltage of <5 V and a high current of 70 mA. Tight distribution of robust Cu pillars for 100 randomly measured devices with an average current of approximately 50 mA at a *V*_read_ of 1 V is observed. The Cu pillars have long read pulse endurance of >10^6^ cycles under positive read voltage. Although, the read pulse endurance under negative read voltage is worst due to Cu dissolution partially. On the other hand, our Al/Cu/Al_2_O_3_/TiN memory device shows good bipolar resistive switching behavior at a CC of 500 μA. Good data retention characteristics of >10^3^ s with acceptable resistance ratio of >10 is observed. It is expected that this novel idea to achieve high-density memory through 3D interconnect will have a good alternative of traditional TSV technique owing to a low cost and simple way.

## Competing interests

The authors declare that they have no competing interests.

## Authors’ contributions

This idea is from SM. RP and DJ fabricated the CBRAM devices under the instruction of SM. RP measured all the devices under the instruction of SM. All authors contributed to the revision of the manuscript. All authors read and approved the final manuscript.
